# Fetal Bovine Serum-Derived Extracellular Vesicles Persist within Vesicle-Depleted Culture Media

**DOI:** 10.3390/ijms19113538

**Published:** 2018-11-09

**Authors:** Brandon M. Lehrich, Yaxuan Liang, Pooya Khosravi, Howard J. Federoff, Massimo S. Fiandaca

**Affiliations:** 1 Translational Laboratory and Biorepository, Department of Neurology, University of California Irvine School of Medicine, Irvine, CA 92697-3910, USA; blehrich@uci.edu (B.M.L.); yaxuan.liang@uci.edu (Y.L.); federoff@uci.edu (H.J.F.); 2 Department of Biomedical Engineering, University of California Irvine, Irvine, CA 92697-3910, USA; pooyak@uci.edu; 3 Department of Information and Computer Sciences, University of California Irvine, Irvine, CA 92697-3910, USA; 4 Department of Neurological Surgery, University of California Irvine School of Medicine, Irvine, CA 92697-3910, USA; 5 Department of Anatomy & Neurobiology, University of California Irvine School of Medicine, Irvine, CA 92697-3910, USA

**Keywords:** fetal bovine serum, nanoparticle tracking analysis, extracellular vesicles, exosomes, astrocyte cell growth, EV-depletion methods

## Abstract

It is known that culture media (CM) promotes cellular growth, adhesion, and protects explanted primary brain cells from in vitro stresses. The fetal bovine serum (FBS) supplement used in most CM, however, contains significant quantities of extracellular vesicles (EVs) that confound quantitative and qualitative analyses from the EVs produced by the cultured cells. We quantitatively tested the ability of common FBS EV-depletion protocols to remove exogenous EVs from FBS-supplemented CM and evaluated the influence such methods have on primary astrocyte culture growth and viability. We assessed two methodologies utilized for FBS EV removal prior to adding to CM: (1) an 18-h ultracentrifugation (UC); and (2) a commercial EV-depleted FBS (Exo-FBS™). Our analysis demonstrated that Exo-FBS™ CM provided the largest depletion (75%) of total FBS EVs, while still providing 6.92 × 10^9^ ± 1.39 × 10^8^ EVs/mL. In addition, both UC and Exo-FBS™ CM resulted in poor primary astrocyte cell growth and viability in culture. The two common FBS EV-depletion methods investigated, therefore, not only contaminate in vitro primary cell-derived EV analyses, but also provide a suboptimal environment for primary astrocyte cell growth and viability. It appears likely that future CM optimization, using a serum-free alternative, might be required to advance analyses of cell-specific EVs isolated in vitro.

## 1. Introduction

Extracellular vesicles (EVs) are lipid-bilayer nanovesicles commonly isolated from both blood plasma and serum [1,2,3], produced by nearly all cell types [4], and secreted into their surrounding extracellular fluid (ECF). Common EV populations found in various biofluids range in size from <50 nanometers (nm; 10^−9^ m) to larger than two microns (>2000 nm) [3,5]. Exosomes, a specific subpopulation of EVs, are produced via an alternative cellular mechanism than other EVs, have a relatively narrow size range (70–120 nm), and contain unique intrinsic cargo molecules (e.g., soluble or membrane bound proteins, lipid species, metabolites, and various nucleic acids) [6,7]. Although all EV subtypes carry distinct cargo molecules consequent to their diverse cells of origin, EVs are also thought to carry an intrinsic capacity to influence both local and/or distant cellular targets [5] through their uptake from the ECF, and providing autocrine-, paracrine-, and endocrine-like influence on target cell biology. 

Various cell types (e.g., primary, cancerous, and induced-pluripotent stem cells [iPSCs]) grown in vitro require a complete (or serum supplemented) culture medium (CM) to optimize growth and viability. This specific supplementation is distinguished from that found in a defined basal medium, such as Dulbecco’s Modified Eagle’s Medium (DMEM), which features basic nutrients for cellular metabolism, proliferation, and homeostasis. A common supplement found in complete CM for optimized cultured cell growth is provided by the addition of heat-inactivated fetal bovine serum (FBS). Differing from what is included in DMEM, the addition of FBS to CM provides additional growth factors and carrier proteins, promotes cell adhesion, and protects cells from stresses associated with the in vitro environment [8]. However, serum in general, and FBS specifically, contains high concentrations of EVs that may also directly influence the cultured cells being nourished in vitro [9,10]. In addition, these FBS-derived EVs “contaminate” any quantitative and/or qualitative assessments of EVs derived from the cultured cells. Under such circumstances, therefore, in vitro experiments designed to explore the biology and characteristics of in vitro-derived EVs necessitates the attempted removal of exogenous EVs from the complete CM, or by utilizing serum-free CM that also supports cultured cell growth and viability. Studies of cultured cell-derived EVs, therefore, have utilized EV-depletion methods in an attempt to eliminate the significant number of FBS-derived EVs found in complete CM, while maintaining other serum factors that support in vitro growth and viability. 

Two major types of FBS EV-depletion approaches have been commonly used, including either (1) high-speed ultracentrifugation (UC), or (2) a proprietary chemical precipitation, commercially available as Exo-FBS^TM^ (System Biosciences, Inc., SBI; Palo Alto, CA). Reports using either of these methods have focused on their respective negative influences on in vitro cellular growth and viability in a variety of cell types [11,12,13,14]. An additional issue, less commonly addressed, is whether the “depleted” supplement added to the CM contaminates any quantitative and/or qualitative analyses of EVs derived from such cultured cells. 

In this manuscript, our aim is to present the quantitative assessment of the EV depletion provided via two commonly used FBS EV-depletion methods, using nanoparticle tracking analysis (NTA). In addition, we also present the differential effects of FBS EV-depletion approaches on the growth and viability of cultured primary astrocytes. It is anticipated that the information presented, and options discussed, will be informative to those planning similar analyses and may ultimately catalyze investigations of relevant cell type-specific EVs in vitro, that may have applicability towards clinical translation.

## 2. Results

### 2.1. An Abundant Population of EVs Remains within EV-Depleted Complete CM

Assessment of our test “untreated” and “depleted” FBS complete CMs, using electron microscopy (EM), provided consistent morphological evidence of included EV populations, similar to those noted in “untreated” and “depleted” human plasma and serum specimens evaluated using UC or ExoQuick™ precipitation (Supplementary Figure S1). Western blot (WB) analyses of EV isolates from human specimens, using density gradient UC, provide evidence (Supplementary Figure S2) of expected exosome/EV markers. NTA analyses of human plasma and serum support the presence of residual exosome and other EV populations following “depletion”. Information gained via preliminary qualitative morphological assessments (EM and WB) and subsequent quantitative analyses (primarily NTA) provided confidence that the latter methodology was measuring residual populations of EVs within our test FBS complete CM specimens. 

**Figure 1:**
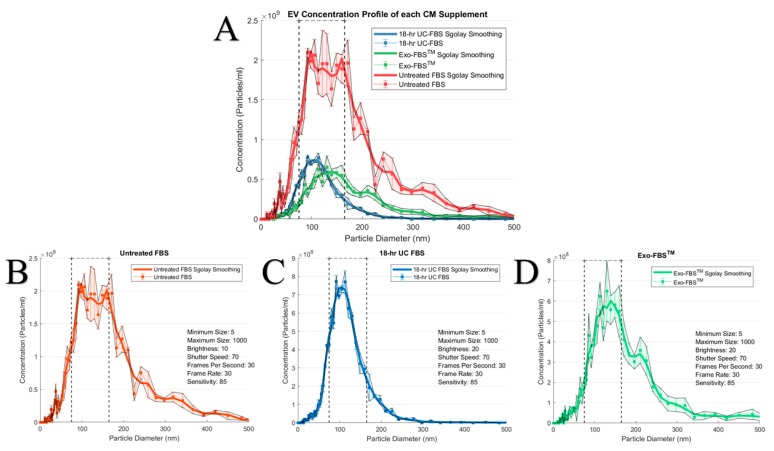
Concentrations and Size Distributions of the Complete Culture Media (CM) Conditions. Untreated fetal bovine serum (FBS)-supplemented complete CM and extracellular vesicles (EV)-depleted FBS-supplemented complete CM (ultracentrifugation (UC) and commercial) were prepared for EV particle analysis using the ZetaVIEW^®^ PMX 110 instrument. Each sample replicate was an independent measure acquired on the instrument (total sample volume of 1 mL) and then grand averaged. Size distribution data was then analyzed and plotted by *BPM ExoPlot*, with filter and smoothing functions, as depicted in the Methods. For all complete CM conditions, graphical representation was set with a “sgolay smooth” smoothing function; this is an algorithm that is a built-in function to MATLAB^®^ signal processing toolbox. Dashed threshold line was defined for EVs with diameters ranging from 75–165 nm. (**A**), Superposition of all three complete CM conditions on the same scaled axes with appropriately adjusted dilution factors to show relative concentration values across each complete CM condition. Individual graphs with detailed measurement parameters are presented for: (**B**), Untreated FBS-supplemented complete CM (*n* = 3 replicates, dilution factor = 1:1000), (**C**), 18-h UC-FBS-supplemented complete CM (*n* = 3 replicates, dilution factor = 1:250), and (**D**), Exo-FBS™-supplemented complete CM (*n* = 3 replicates, dilution factor = 1:333.33).

A superposition of each test complete CM’s EV population size distribution, derived via NTA, on the same set of axes, depicts the incomplete FBS EV “depletion” in the test CM conditions compared to the untreated complete CM (Figure 1A). Untreated FBS-supplemented complete CM contains a heterogeneous population of EVs, with sizes ranging from <50 nm to >500 nm (Figure 1B). After an 18-h UC of the FBS, a large population of EVs persists within supernatant added to supplement the CM (Figure 1C), especially compared to the same scale assessments evaluating the Exo-FBS™-supplemented complete CM (Figure 1D). Furthermore, when profiling the size distributions of the EV populations in the two “depleted” complete CM conditions, the 18-h UC-FBS supplemented complete CM provided the best depletion of larger, non-exosomal (>150 nm) EVs, with the majority of the remaining EVs having mean diameters ranging from 75–250 nm (Figure 1C), while those remaining within the Exo-FBS™-supplemented complete CM had mean diameters that ranged from 75–500 nm (Figure 1D). 

**Figure 2:**
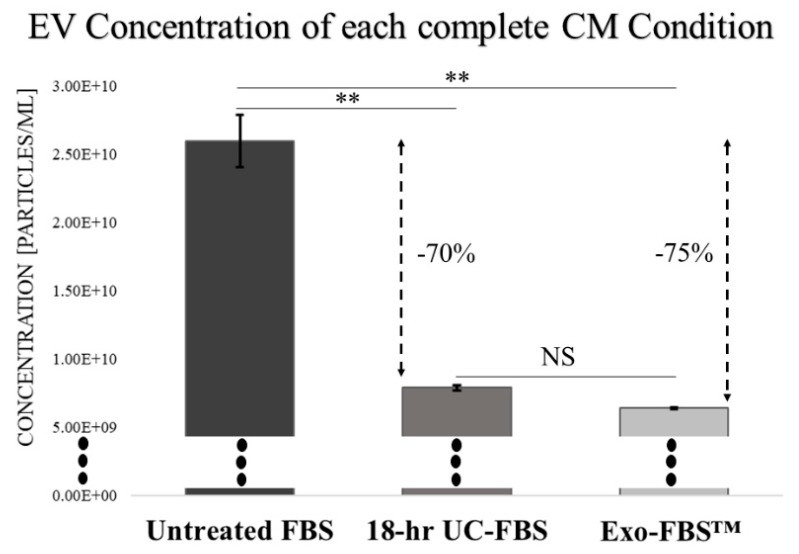
Relative Magnitudes of defined EV Concentrations for each Complete CM Condition. The defined EV population with a common size ranging from 75–165 nm was extracted, together with the mean total EV concentration values across all complete CM conditions to illustrate relative depletion (*n* = 3 replicates). Exo-FBS™-supplemented complete CM provided the lowest statistically significant EV concentration, although not significantly different from the 18-h UC-FBS-supplemented complete CM (p = 0.99). ** *p* < 0.01. NS = no statistical significance.

Relative concentrations of EVs within a common size range (75–165 nm) were defined for the different complete CM conditions (vertical dashed line thresholds shown in Figure 1). The 10% untreated FBS-supplemented complete CM provided a concentration of 2.60 ± 0.33 × 10^10^ EVs/mL in the common range. In contrast, the 10% 18-h UC-FBS-supplemented complete CM concentration was 7.90 ± 0.34 × 10^9^ EVs/mL, and the 10% Exo-FBS™-supplemented complete CM concentration provided 6.41 ± 0.14 × 10^9^ EVs/mL (Figure 2). When compared to the untreated FBS-supplemented complete CM, the 18-h UC-FBS-supplemented complete CM provided a 70% EV depletion, and the Exo-FBS™-supplemented complete CM a 75% EV depletion. 

### 2.2. Astrocyte Growth and Viability Is Reduced in EV-Depleted Serum Conditions

**Figure 3:**
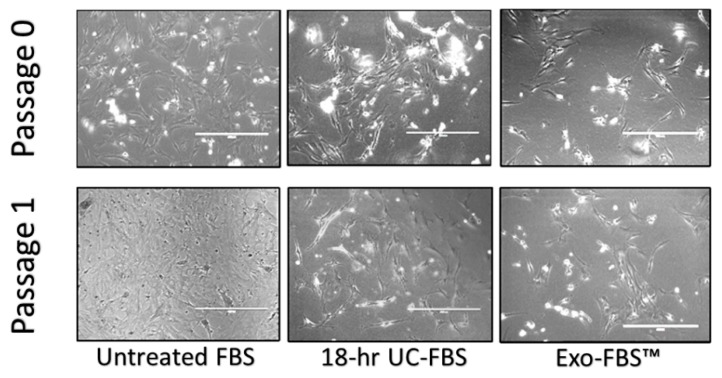
Primary Astrocyte Morphological Growth Differences Across Two Passage Time Points. Photomicrographs were obtained using an EVOS™ Fluorescent Cell Imaging System after each passage time point (approximately 3–5 days, for each of the three distinct complete CM conditions), scale bar = 400 µm.

Utilizing both immunocytochemistry (ICC) and fluorescence-activated cell sorting (FACS), the remaining adherent cells in our T25 flasks were found to be >85% GFAP positive (data not shown) and will be considered astrocytes. We observed qualitative astrocyte morphological differences with our test complete CM conditions (Figure 3). Differences in cultured primary astrocytes with EV-depletion methods included (a) decreases in size of the cell body, (b) increased appearance of floating or dead astrocyte profiles, and (c) the presence of curved and detached, but viable cells. A DNA laddering assay and flow cytometry was performed to investigate the potential for apoptosis/necrosis across the EV-depleted complete CM conditions. The results were inconclusive (data not shown) and require future focused analyses. Additionally (Figure 3), while 100% cell confluence was observed when using untreated FBS-supplemented complete CM over one passage period (Passage 0 to Passage 1 = 3–5 days), only about 70% confluence was documented during this same interval when using the 18-h UC-FBS-supplemented complete CM, and only 50% confluence was noted using the Exo-FBS™-supplemented complete CM. 

**Figure 4:**
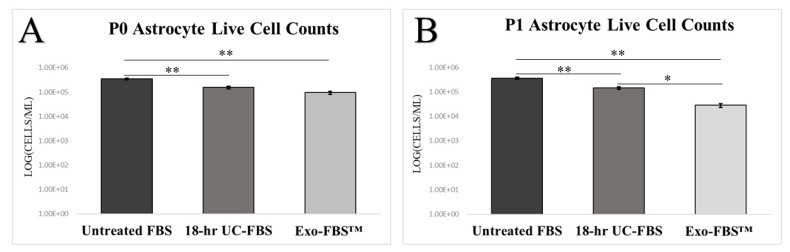
Quantitative Primary Astrocyte Viability Across Complete CM Conditions. Live cell counts for each complete CM condition (Untreated FBS-supplemented complete CM [*n* = 6], 18-h UC-FBS-supplemented complete CM [*n* = 6], Exo-FBS™-supplemented complete CM [*n* = 6]) were obtained using a BioRad TC20™ Automated Cell Counter. Error bars represent standard error of the mean (SEM). ** *p* < 0.01. * *p* < 0.05.

We also quantified cell viability over two passage time points across all complete CM test conditions. Our comparative quantitative analyses revealed that after the second cell passage, significant differences in live cell counts were noted between the untreated FBS-supplemented complete CM and 18-h UC-FBS-supplemented complete CM (*p* < 0.01), and between untreated FBS-supplemented complete CM and Exo-FBS™-supplemented complete CM (*p* < 0.01) (Figure 4A). Additionally, significant increases in live cell counts existed between 18-h UC-FBS-supplemented complete CM and Exo-FBS™-supplemented complete CM (*p* < 0.05), with the 18-h UC-FBS-supplemented complete CM providing improved cell viability compared to using the Exo-FBS™-supplemented complete CM (Figure 4B). 

## 3. Discussion

This study provides quantitative evidence of the incomplete depletion of FBS-derived EVs within the complete CM that has undergone presumed “depletion” using two common FBS-EV depletion methodologies. In addition, both depletion methods investigated provide a negative influence on primary astrocyte growth and viability in vitro. A failure in FBS EV-depletion is especially relevant to those investigating endogenously produced EVs (e.g., exosomes) in vitro by specific cell types. Although prior publications have also described the effects of FBS EV-depletion methods regarding cellular growth and viability, organelle stress, and altered gene regulation [12,14], an additional concern advancing our work was regarding whether the depletion of exogenous FBS-derived EVs was adequate to prevent interference of in vitro analyses of EVs produced by primary cells. We believe that exogenous EVs present within the FBS supplement contaminate results provided for any bioengineering, therapeutic, and/or diagnostic investigations focused only on endogenously derived EVs produced by the cultured cells [15,16]. 

We currently lack a full appreciation for the final makeup of the FBS pellet produced by either the 18-h UC or Exo-FBS^TM^ precipitation procedures. We infer that they likely include an incomplete population of FBS-derived EVs, in addition to a variety of proteins and other precipitant species. The incomplete removal of lipid bilayer nanovesicles from FBS makes it extremely difficult to claim any in vitro cell-specific biomolecular profiling of EVs, especially when utilizing complete CM, even if derived using the two described FBS EV-depletion methods. This latter point was recently illustrated in an article [16] that found many of the FBS-derived RNA transcripts, both free-floating species and within EVs, map to both the human and mouse genomes, leading prior investigations towards the inadvertent false-positive reporting of extracellular RNA release from cultured cells. 

In addition, previous reports providing FBS EV-depletion analyses, using either dynamic light scattering (DLS) and/or NTA, have lacked protocol standardization and reproducibility across instruments and laboratories. Specifically, such analytic methods pose distinct issues, such as: (1) DLS, NTA, and resistive pulse sensing (RPS), hold a limit of detection to be at 70–80 nm [17], thereby questioning the ability to define and quantify EV populations at the sub-50 nm range of accuracy; and (2) standardization of DLS, NTA, and RPS instrumentation requires reporting of not only dilution factors, but also specific instrument settings (for consistency across multiple sample replicates) that aim to accurately define and replicate the total number of EV-like particles found in a suspension [18]. In our study, all quantitative analyses of EVs using our NTA device were limited to the 75–165 nm range, while maintaining consistent and reproducible instrument settings between sample runs and adjusting only the sample dilution prior to analyses for optimization of instrument readings and reliability. 

Moreover, defining “pure” EV fractions has become an important topic in the field and has led to the establishment of recommended guidelines from the International Society for Extracellular Vesicles (ISEV) for characterizing the final EV isolate from cultured cells [19]. However, the guidelines have lacked the inclusion of criteria to confirm the lack of FBS EV cross contamination within the complete CM used for isolation of cultured cell-derived EVs. In fact, many publications [20,21] detailing in vitro exosome release characteristics have yet to quantify the residual EVs within the complete CM used for exosome (and EV) collection and subsequent analyses. The first step in adequately isolating “pure” cultured cell-derived EV fractions should be the inclusion of media that is completely devoid of FBS (or other extraneous) EV populations. 

We believe, therefore, that it remains relevant for investigators to consider alternative complete CM formulations, beyond using the two common FBS depletion protocols we investigated, for any in vitro studies featuring cell-derived EV analyses. A novel methodology, ultrafiltration [22], may provide such an alternative, through the use of a 100 kDa molecular weight membrane filter that removes FBS-derived EVs from complete CM used for in vitro EV studies. More functional assays (e.g., nucleic acid sequencing, protein quantification, and robust nanoparticle tracking analytics) using these and other methodologies, however, need to be thoroughly vetted prior to achieving widespread adoption. Finally, an evolving, but seemingly viable option utilizes serum-free CM, providing no exogenous EVs to the in vitro environment [23]. The latter method has yet to define the ideal additives for a complete CM recipe necessary for cell growth and viability approaching that of FBS-supplemented complete CM for use with all cell types in vitro. The benefits of such an engineered serum-free CM may include a better appreciation of individual cultured cell physiology and pathobiology, and how the specific addition of external influences (e.g., EVs) might influence cell biology. Understanding the quantities (concentrations) of such “specifically-engineered supplements” required for proper cell growth and phenotypic function are essential to defining a future serum-free CM option that is devoid of exogenous EVs. Such an option is likely to yield an improved understanding of the genesis of EVs directly from cultured cells, with delineation of their cell- and phenotype-specific cargos, and eventually lead to an advanced diagnostic [24] as well as novel therapeutic approaches for maintaining human health. 

## 4. Materials and Methods

### 4.1. Preparation of EV-Depleted Serum for Testing

**Figure 5:**
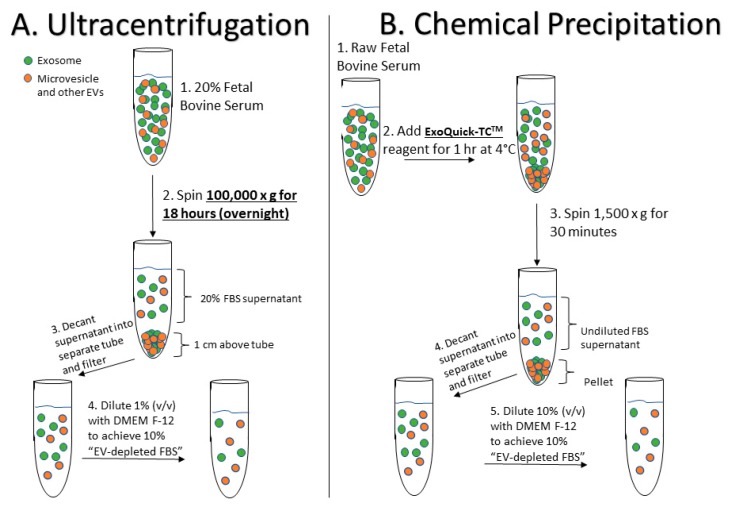
Fetal Bovine Serum EV-Depletion Methodologies Schematic. (**A**) 18-h ultracentrifugation (UC) approach to EV-depletion. (**B**) As per the System Biosciences, Inc. (SBI) patent for Exo-FBS^TM^ (US 20130337440A1), the commercial process includes chemical precipitation using ExoQuick-TC™ reagent and filtration steps.

The main FBS EV-depletion protocols utilized in this study are depicted in Figure 5. In preparation for this study we gained experience performing similar “depletion” analyses on human serum and plasma specimens, whose EV populations were qualitatively assessed via electron microscopy (EM) and western blot (WB) analyses (see Supplementary Figures S1 and S2), and quantified using NTA. For this study, untreated FBS was prepared by diluting heat-inactivated FBS (Thermo Fisher, MA, USA; Cat #10438-026) in basal culture media (CM; DMEM-F12 and 1% penicillin streptavidin [Thermo Fisher, MA, USA]; Cat #11320-033) to reach a final concentration of 10% FBS. 18-h UC-FBS (Figure 1A) was prepared by first diluting FBS in CM to produce a 20% FBS concentration (*v*/*v*), and then centrifuging overnight (approximately 18 h) at 100,000× *g* at 4 °C, using a SW41 Ti Rotor (k factor = 124) in a Beckman Coulter Optima^TM^ L-100 XP Ultracentrifuge. The resulting supernatant was then carefully removed via pipet, making sure not to disturb the pelleted material at the bottom of the UC tube (approximately 1 cm above the bottom). After passage through a 0.22-μm filter (Millex No. SLGP033RS), the UC supernatant was further diluted with CM (1:2) to achieve a final 10% concentration of EV “depleted” FBS. Finally, commercial Exo-FBS™ (Figure 1B) supplement was purchased from System Biosciences, Inc. (SBI, Palo Alto, CA, USA), passed through a 0.22 µm filter, and finally diluted (to 10% *v*/*v*) in CM. 

### 4.2. Primary Rat Astrocyte Cell Cultures

Sprague Dawley postnatal day 2 (P2) rats provided cerebral cortices for primary neural cell explants. All brain harvests were carried out in accordance with the Institutional Animal Care and Use Committee at the University of California, Irvine, and were consistent with Federal guidelines. Primary rat astrocyte cultures were derived using previously published methods [25]. Briefly, ten fresh cerebral cortices were collected and dissociated into a single cell suspension in DMEM. Cells suspensions were counted and then plated into T25 flasks (Thermo Fisher Scientific, Waltham, MA, USA) at a density of 2.5 × 10^6^ cells/mL, using 10% untreated FBS-supplemented complete CM. Culture flasks were maintained in an incubator, at a temperature of 37 °C and under 5% CO_2_. Forty-eight (48) hours after the initial plating, the sides of the flasks were gently tapped to dislodge contaminating, non-adherent cell-types (i.e., neurons, microglia, and oligodendrocytes) from the more adherent astrocyte bed layer. A full media exchange with FBS-supplemented complete CM was then performed to remove the dislodged cell types. ICC and FACS was performed using an anti-GFAP antibody (Santa Cruz Biotechnology, Inc., Dallas, TX, USA; Cat # sc-166458), thereby confirming the identity of cultured astrocytes. Cells adherent to the T25 flasks were then grown and split (1:4) at near-confluence into 12-well plates (seeding ~7.5 × 10^4^ cells/mL), using glass coverslips to allow monitoring of cell growth and viability under each complete CM test condition. 

### 4.3. EV Size and Concentration Determinations via Nanoparticle Tracking Analytics

The ZetaVIEW**^®^** PMX 110 (Particle Metrix, Meerbusch, Germany) NTA instrument provided the gauss (or log normal) size distribution of the EV populations found in the test complete CM conditions [26]. Polystyrene nanoparticle standards (102 nm; ThermoFisher Scientific Inc., Waltham, MA, USA [Cat: #3100A]) allowed instrument calibration prior to each day’s analyses. Test complete CM sample aliquots were serially diluted in phosphate-buffered saline to provide optimal initial ZetaVIEW**^®^** instrument readings (10^6^–10^9^ particles/mL), and then evaluated for consistency over three measurement cycles. Sample readings were determined using a set of standardized ZetaVIEW**^®^** pre-acquisition parameters: temperature of 23 °C; sensitivity of 85; video frame rate of 30 frames per second; and, a capture shutter speed of 100. Post-acquisition ZetaVIEW**^®^** instrument parameters for EV species were defined during the analysis and included: minimum brightness of 25; maximum size of 200 pixels; and, a minimum size of 5 pixels (with pixel size not correlating to an equivalent nanometer diameter value). The ZetaVIEW**^®^** instrument provides a text (.txt) file that includes the mean, median, and mode of visualized nanoparticle (EV) diameters, as well as the relative EV concentration (particles/mL) within the sample (based on an input dilution measure of the original sample). These data from the ZetaView**^®^** were analyzed using a proprietary software package (ZetaView**^®^** 8.02.28) and graphically displayed and further analyzed via a custom MATLAB**^®^** File Exchange Application (*BPM ExoPlot*) [27]. 

### 4.4. Cell Counts and Time-Lapse Photo-Microscopy

Daily differential in vitro cell growth was qualitatively monitored using an EVOS™ FL Color (Cat #AMEFC4300) Cell Imaging System (ThermoFisher Scientific Inc, Waltham, MA, USA) over the course of each passage interval. Full media exchanges with each well’s respective complete CM condition occurred every 48 h. After the cells reached 80–90% confluence, as visualized and documented via photomicroscopy, adherent cells were trypsinized, collected, pelletized, and evaluated for cell viability. Cell viability was measured using the Bio-Rad TC20™ Automated Cell Counter (Bio-Rad, Hercules, CA, USA) using established methods [28], providing total and live cell counts for each complete CM condition. The content from each culture well, corresponding to a different complete CM condition, was collected, and counted. Replicate samples were evaluated individually and not pooled. 

### 4.5. Statistics 

Statistical analyses were performed using PASW Statistics 18.0 software (SPSS Inc, Chicago, IL, USA) with significance set at a value of *p* < 0.05. One-way analysis of variance (ANOVA) with Tukey HSD post-hoc corrections were used to compare continuous variables between group means. 

## 5. Conclusions

The current study reveals that available FBS EV-depletion methods fail to provide adequate reductions in FBS-derived EV populations within “depleted” complete CM. In addition, there are apparent negative effects on cultured primary astrocyte growth and viability using both tested “depletion” methods. We conclude, therefore, that the commonly used methods (ultracentrifugation and Exo-FBS™) used to produce EV-depleted complete CM remain inadequate for quantitative assessments of EVs produced in vitro and for providing optimal primary cultured cell growth and viability. With the latter result, our study suggests that other soluble factors, precipitated and/or removed from the FBS during the two tested depletion processes, influence primary astrocytes’ ability to thrive in culture. There remains a great need for an engineered CM, completely devoid of exogenous EVs, that supports cell growth and viability while eliminating the likelihood of exogenous EVs influencing the phenotype and EV production of a primary cultured cell. The development of such a complete CM is likely to catalyze investigations of specific cell–derived EVs (and their cargos) derived from primary cell cultures. Such details will advance our understanding of cell biology and ultimately EV-associated functions within complex biofluids.

## Supplementary Materials

Supplementary materials can be found at http://www.mdpi.com/1422-0067/19/11/3538/s1. Figure S1: Transmission Electron Microscopy Whole Mount Slide of Human Plasma Extracellular Vesicles (EVs). Figure S2: Western Blot (WB) of Human Plasma EVs.

## Abbreviations

CM	Culture Media
FBS	Fetal Bovine Serum
EV	Extracellular Vesicle
ECF	Extracellular Fluid
iPSCs	Induced-Pluripotent Stem Cells
DMEM	Dulbecco’s Modified Eagle’s Medium
UC	Ultracentrifugation
NTA	Nanoparticle Tracking Analytics
ICC	Immunocytochemistry
FACS	Fluorescence-Activated Cell Sorting
DLS	Dynamic Light Scattering
RPS	Resistive Pulse Sensing
ANOVA	One-way Analysis of Variance
