# Supplementary material for: Fetal Bovine Serum-Derived Extracellular Vesicles Persist within Vesicle-Depleted Culture Media
Source: Int J Mol Sci. 2018 Nov 9;19(11):3538. doi: 10.3390/ijms19113538 (PMC6275013; doi:10.3390/ijms19113538)
Supplement: Supplementary file 1 [file ijms-19-03538-s001.pdf]

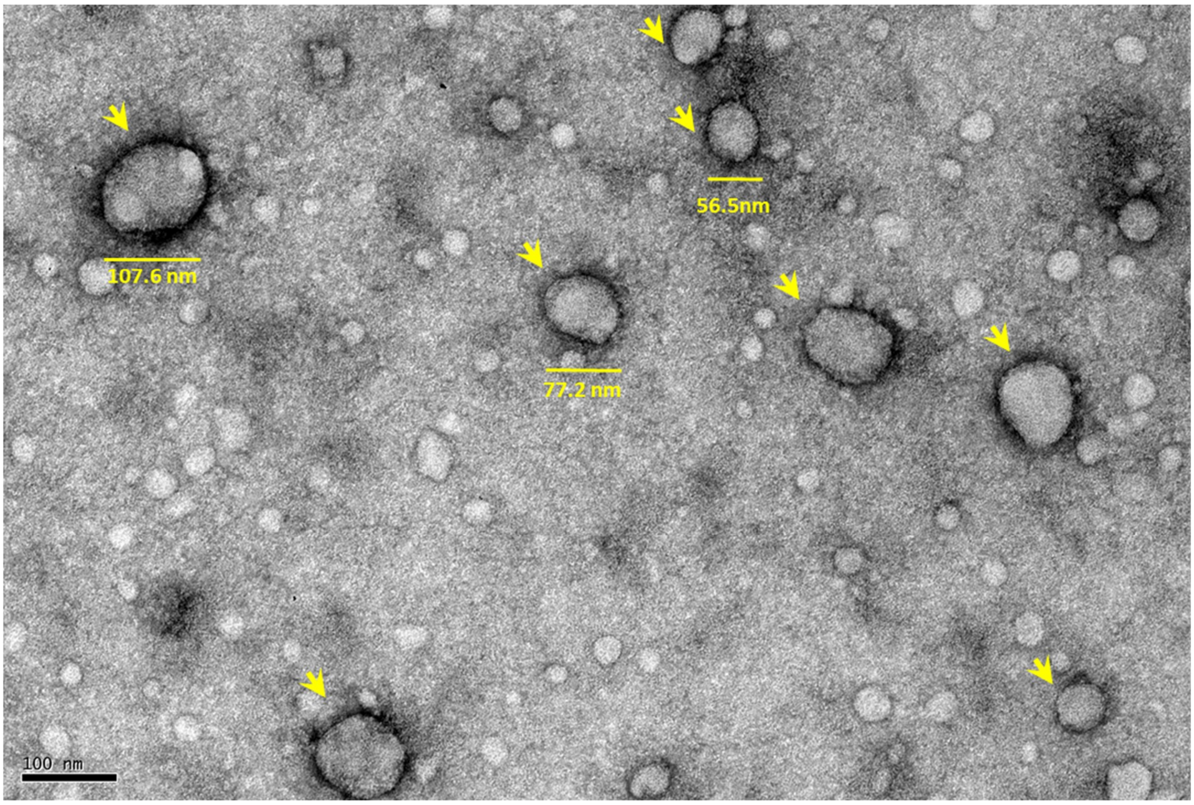


**Figure S1**: Transmission Electron Microscopy Whole Mount Slide of Human Plasma Extracellular Vesicles (EVs). A human plasma EV pellet was obtained following high-speed ultracentrifugation (UC) at 120,000 ×*g* for 2 h, from a 500 L aliquot. Plasma nanoparticle tracking analysis (NTA) data, before and after UC, documented quantitative reductions in EV populations following UC "depletion" (unpublished data). The plasma EVs were fixed with 2% paraformaldehyde and negatively stained with 2% uranyl-acetate for qualitative visualization using transmission electron microscopy (TEM) (scale bar = 100 nanometers [nm]). Note the significant number of visible nanovesicles in the field with apparent diameters <50 nm. Size estimates for certain vesicles are provided in yellow numerals. Nanovesicles indicated by yellow arrows are in the size range compatible with exosomes.

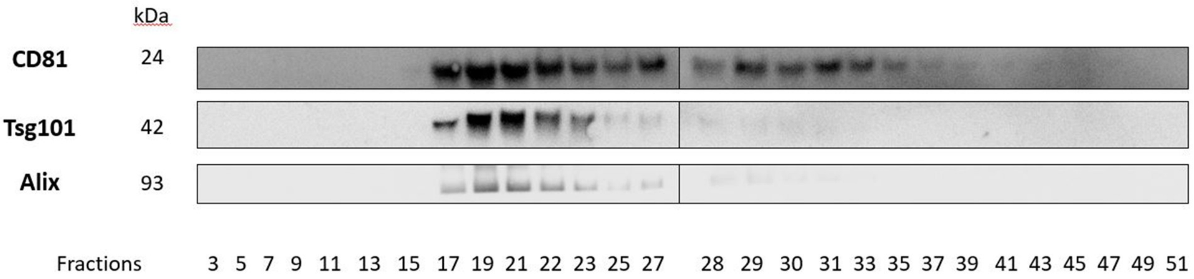


**Figure 2**: Western Blot (WB) of Human Plasma EVs. A 500 L aliquot of human plasma was layered on top of a stepwise iodixanol density gradient and underwent high-speed ultracentrifugation (120,000 ×*g* for 18 h). Representative concentration fractions are depicted on the WB, from low to high density (fraction 3 to 51, respectively). Canonical exosome and/or EV markers (e.g., CD81, Tsg101, and Alix) were

visualized in each fraction using marker-specific human antibodies. All three makers appear robustly enriched within fractions 19–22, while less so in other fractions.
